# Whole genome characterization of feline coronaviruses in Thailand: evidence of genetic recombination and mutation M1058L in pathotype switch

**DOI:** 10.3389/fvets.2025.1451967

**Published:** 2025-02-14

**Authors:** Eaint Min Phyu, Kamonpan Charoenkul, Chanakarn Nasamran, Ekkapat Chamsai, Yu Nandi Thaw, Hnin Wai Phyu, Han Win Soe, Supassama Chaiyawong, Alongkorn Amonsin

**Affiliations:** ^1^Center of Excellence for Emerging and Re-emerging Infectious Diseases in Animals, Faculty of Veterinary Science, Chulalongkorn University, Bangkok, Thailand; ^2^The International Graduate Course of Veterinary Science and Technology, Chulalongkorn University, Bangkok, Thailand; ^3^Department of Veterinary Public Health, Faculty of Veterinary Science, Chulalongkorn University, Bangkok, Thailand

**Keywords:** cats, characterization, feline coronavirus, whole genome sequence, Thailand

## Abstract

Feline coronavirus (FCoV) is a significant pathogen that infects the feline population worldwide. FCoV can cause mild enteric disease and a fatal systemic disease called feline infectious peritonitis (FIP). In this study, a cross-sectional survey of FCoV in domestic cats from small animal hospitals in Thailand was conducted from January to December 2021. Our result showed that out of 238 samples tested for FCoV using 3’ UTR-specific RT-PCR, 18.7% (28/150) of asymptomatic cats and 25.5% (12/47) of cats with unknown status tested positive for FCoVs. Additionally, 51.2% (21/41) of cats with suspected FIP were found to be positive for FCoVs. Genotype identification using S gene-specific RT-PCR showed that all FCoV-positive samples (n = 61) were FCoV type I. This study obtained the whole genome sequences (*n* = 3) and S gene sequences (*n* = 21) of Thai-FCoVs. Notably, this study is the first to report the whole genome of Thai-FCoV. Phylogenetic analysis indicated that Thai-FCoVs were closely related to FCoVs from China and Europe. Additionally, the Thai-FCoVs exhibited specific amino acid substitutions (M1058L) associated with the pathotype switch. Recombination events were found to mainly occur in the ORF1ab and S gene regions of Thai-FCoVs. This study provides insights into the occurrence, genetic diversity, virulence amino acid mutations, and potential recombination of FCoVs in the domestic cat population in Thailand, contributing to our understanding of FCoV epidemiology.

## Introduction

Feline coronavirus (FCoV) is an enveloped, non-segmented, single-stranded RNA virus that belongs to the family *Coronaviridae* and genus *Alphacoronavirus*. The viral genome consists of 11 open reading frames (ORFs) and encodes 11 proteins: Replicase 1a and 1b polyproteins (1ab), Spike (S), Envelope (E), Matrix (M), Nucleocapsid (N), ORF3abc and 7ab (1, 2). FCoVs can be classified into two pathotypes, feline enteric coronavirus (FECV) and feline infectious peritonitis virus (FIPV), based on the pathogenicity (3). FECV usually leads to mild enteritis, while FIPV causes a systemic lethal disease known as feline infectious peritonitis (FIP) (4, 5). Feline Infectious Peritonitis (FIP) is usually characterized by protein-rich serous effusion in the body cavity and granulomatous inflammatory lesions in the internal organs (6). The switch from FECV to FIPV is not well understood, but previous studies suggested that mutations in the FECV genome may be responsible for the emergence of FIPV (3, 7–9). FCoV can also be genetically classified into two genotypes, FCoV type I and FCoV type II, based on structural differences in the S gene (10). Among the two genotypes, FCoV type I is more predominant than FCoV type II worldwide. While FCoV type II originated from a recombination between FCoV type I and canine coronavirus (CCoV) (11). Both FCoV genotype I and genotype II can cause FIP (12–15).

In Thailand, the first report of FCoV was documented in 2003 (16). It was documented that FCoV type I had a higher prevalence than FCoV type II, and mixed infection of both genotypes was also found (15). The sequence analysis on the ORF3abc, E, M, N, and 7ab genes of Thai-FCoVs was also documented (17). Currently, there are only 120 nucleotide sequences of certain genes (3abc, 7ab, S, E, M, N) of Thai-FCoVs available in the GenBank database. However, information on the whole genome and completed S gene sequences of Thai-FCoVs is still limited. Therefore, in this study, we conducted a cross-sectional survey of FCoV and characterized the whole genome, and completed S gene of Thai-FCoVs to obtain information on the genetic diversity of circulating Thai FCoVs. This study is the first to report the whole-genome characterization of Thai-FCoVs.

## Materials and methods

### Sample collection from domestic cats

In this study, we conducted a cross-sectional sample collection from 12 animal hospitals in Bangkok and the vicinity from January to December 2021. A total of 238 samples were collected from cats during hospital visits, including 197 rectal swab samples and 41 abdominal/thoracic fluid samples. The samples were collected from Bangkok (*n* = 133), Nonthaburi (*n* = 80), and Samut Prakan (*n* = 25). In this study, we collected rectal swab samples from asymptomatic cats (*n* = 150) and cats with unknown status (*n* = 47) (cats with various clinical signs, e.g., fever, diarrhea, vomiting, anorexia, coughing, and chronic diseases). We also acquired abdominal and/or thoracic fluid samples (*n* = 41) from cats suspected to have FIP, which developed effusions in the body cavity. Data related to the collection date, age, breed, sex, and clinical status of all animals were recorded. This study was conducted with approval from the Institute of Animal Use and Care Committee (IACUC# 2331076), and all procedures were completed in accordance with the relevant guidelines and regulations.

### Molecular detection of FCoV

The samples, including rectal swab samples (*n* = 197) and fluid samples (*n* = 41), were subjected to RNA extraction using a magnetic bead-based automatic purification equipment of GENTi^™^ 32—Automated Nucleic Acid Extraction System (GeneAll^®^, Seoul, South Korea). Briefly, the rectal swab sample was vortexed for at least 15 s before removing the swab. Next, 7 μL of RNA carrier was added to the extraction tube, and 200 μL of sample was then added to the same tube. The RNA extraction was carried out according to the manufacturer’s instructions. The extracted RNA was kept at −20°C until testing.

For the detection of FCoV, one-step RT-PCR was performed using primers targeting the 3’UTR region of the FCoV genome (10). Briefly, one-step RT-PCR was conducted in a total final volume of 25 μL comprised of 3 μL of template RNA, 1x reaction mix, 0.25 μM of each forward and reverse primer, 20 units of SuperScript III RT (Invitrogen, United States) and distilled water. The RT-PCR conditions included a cDNA synthesis step at 55°C for 30 min, an initial denaturation step at 94°C for 2 min, 40 cycles of denaturation at 94°C for 15 s, annealing at 55°C for 30 s, extension at 68°C for 30 s, and a final extension step at 68°C for 5 min. To confirm FCoV, 3 μL of PCR product was run on a 1.5% agarose gel with red safe. The expected size of the positive FCoV product was 223 base pairs.

### Genotype identification and whole genome sequencing of FCoV

All FCoV-positive samples (*n* = 61) were subjected to genotype identification using S gene-specific RT-PCR as previously described (18). The PCR condition included a cDNA synthesis step at 55°C for 30 min, an initial denaturation step at 94°C for 2 min, 40 cycles of denaturation at 94°C for 15 s, annealing at 55°C for 30 s, extension at 68°C for 30 s, and a final extension step at 68°C for 5 min. The expected product size for FCoV type I and FCoV type II is 360 bp and 238 bp, respectively.

After genotyping, we selected 24 representative FCoV-positive samples based on the based on the date, location, and clinical status of the animal for whole genome and S gene sequencing. First, the RNA was subjected to cDNA synthesis with random hexamers using SuperScript™ IV Reverse Transcriptase (Thermo Fisher Scientific) according to the manufacturer’s instructions. After cDNA synthesis, the whole genome and S gene sequencing were performed using newly designed specific primers by Primer 3 plus and primer sets described previously (Supplementary Table S1) (19). The protocol used to amplify all ORFs of FCoV with Platinum^™^ Taq DNA Polymerase High Fidelity (Thermo Fisher Scientific) was performed according to the manufacturer’s instructions. The PCR conditions included an initial denaturation step at 94°C for 30 s, 40 cycles of denaturation at 94°C for 15 s, an annealing step depending on the primer Tm for 30 s, and an extension step at 68°C for 1–2 min. A final extension step at 68°C for 7 min was included. The amplified PCR products were then pooled together and purified using NucleoSpin^®^ Gel and PCR Clean-up (MACHEREY-NAGEL^™^, Germany) according to the manufacturer’s instructions. The whole genome and S gene sequencing were performed using an Oxford Nanopore rapid sequencing kit (SQK-RAD004) according to the manufacturer’s instructions. In detail, first, to prepare the DNA library, 3.75 μL of purified PCR product was mixed with 1.25 μL of fragmentation mix (FRA) and incubated in a thermocycler at 30°C for 1 min, then at 80°C for 1 min, and finally cooled on ice. After that, 0.5 μL of rapid adapter (RAP) was added and incubated at 25°C for 5 min. To complete the DNA library loading, 15 μL of sequencing buffer (SQB), 10 μL of loading beads (LB), and 5 μL of nuclease-free water were added. The flow cell priming mix was prepared by the mix of 3 μL flush tether (FLT) with 117 μL of flush buffer (FB). For sequencing, 200 μL of flow cell priming mix was firstly loaded to the flow cell, and then 30 μL of prepared DNA library was followed, and the sequencing process was started through MinKNOW software. After sequencing, MinKNOW software was used to convert the data from Fasta 5 file format to Fastq file format. A minimum Q-score of 7 was used to filter out the low-quality sequences. The nucleotide sequences were assembled using de-novo assembly with Genome Detective web software^1^ and Qiagen CLC Genomics Benchwork version 20.0.4 software (QIAGEN, CA, United States).^2^ In this study, we have accomplished three whole genome sequences, nine complete S gene sequences, and twelve partial S gene sequences. The nucleotide sequences were submitted to the GenBank database under the accession # PP901870-PP901890 and PP908788-PP908790.

### Phylogenetic and genetic analyses of Thai-FCoVs

The phylogenetic analysis involved comparing nucleotide sequences of each gene of Thai-FCoVs with reference nucleotide sequences of CoVs from the GenBank database. The reference CoVs were chosen based on their different geographical distributions and hosts. The phylogenetic trees were constructed using MEGA v.11.0.11 software with a neighbor-joining method and Kimura 2-parameter with 1,000 bootstrap replications (20). Thai-FCoV sequences from this study and reference CoV sequences were aligned using the ClustalW function. For each gene analysis, the nucleotide sequences of each gene of FCoV were used to generate the phylogenetic tree of the virus using MEGA v.11.0.11 software. For pairwise comparison, nucleotide sequences and amino acids of Thai-FCoVs were aligned with those of reference CoVs, and calculated using the pairwise distance function in MEGA v.11.0.11 software. For genetic analysis, deduced amino acids of Thai-FCoVs were aligned with those of reference CoVs using MegAlign version 5.03 (DNASTAR Inc., Madison, WI, United States) software.

### Recombination analysis of Thai-FCoVs

RDP5 and Simplot v.3.5.1 programs were used to detect potential recombination events of Thai-FCoVs in this study. Only whole genome sequences were used for analysis. In brief, potential recombination events were identified using the RDP5 program, which included different methods with an acceptable *p-value* of 0.05 (21). Subsequently, the potential recombination breakpoints were further identified using the Simplot v.3.5.1 program using the bootscan function with the neighbor-joining model, 1,000 bootstrap replicates, and Kimura (2-parameter) distance model. A sliding window of 1,000 nucleotides and 200 nucleotide steps was used as the default settings (22).

### Statistical analysis

The association of FIP-related clinical presentations and amino acid substitution at positions 1,058 and 1,060 of the fusion protein in S protein was performed using the chi-square test in IBM SPSS Statistics, version 29.0.1.0. A *p-value* of < 0.01 was considered statistically significant.

## Results

From January to December 2021, a total of 238 samples from domestic cats, including 197 rectal samples and 41 abdominal/thoracic fluid samples, were collected from 12 small animal hospitals in Bangkok, Nonthaburi, and Samut Prakan. In this study, the domestic cats were categorized into three groups based on their clinical status: asymptomatic cats, cats with unknown status (sick cats with general clinical signs), and FIP-suspected cats (cats with clinical signs and the development of effusion in the body cavity). The 238 samples were tested for FCoV using RT-PCR specific to the 3’UTR region, and 18.7% (28/150) of asymptomatic cats and 25.5% (12/47) of cats with unknown status tested positive for FCoVs. Meanwhile, 51.2% (21/41) of FIP-suspected cats were found positive for FCoVs (Table 1). The positive rates of FCoV in Bangkok, Nonthaburi, and Samut Prakan were 32.3% (43/133), 16.3% (13/80), and 20.0% (5/25), respectively. Cats younger than six months were more likely to be infected with FCoV (41.30%, 19/46) than adults and older, with a statistically significant (*p-value < 0.01*). While FCoV infection was slightly higher in male cats, 26.0% (32/123) than in female cats, but it was not statistically significant. Moreover, the FCoV positive rate was the highest in winter, with 29.51% (18/61) (Supplementary Table S2).

**Table 1:** List of samples collected and FCoV identification by location, age, sex, season, and clinical presentations in this study.

	FCoV identification, FCoV positive/sample tested (% positive)			
	Asymptomatic*	FIP-suspected**	Unknown status***	Total
Location				
Bangkok	18/79 (22.8)	19/35 (54.3)	6/19 (31.6)	43/133(32.3)
Nonthaburi	9/60 (15.0)	1/3 (33.3)	3/17 (17.6)	13/80 (16.3)
Samut Prakan	1/11 (9.1)	1/3 (33.3)	3/11 (27.3)	5/25 (20.0)
Age				
Young cats (<6 months)	7/24 (29.2)	6/8 (75.0)	6/14 (42.9)	19/46 (41.3)
Older cats (>6 months)	15/80 (18.8)	3/11 (27.3)	4/23 (17.4)	22/114 (19.3)
N/A	6/47 (12.7)	12/22 (54.5)	2/9 (22.2)	20/78 (25.6)
Sex				
Male	17/74 (22.9)	8/22 (36.4)	7/27 (25.9)	32/123 (26.0)
Female	10/65 (15.4)	4/7 (57.1)	5/17 (29.4)	19/89 (21.3)
N/A	1/11 (9.1)	9/12 (75.0)	0/3 (00.0)	10/26 (38.5)
Season				
Summer (Mar-May)	12/58 (20.7)	0/0 (00.0)	2/15(13.3)	14/72 (19.4)
Rainy (Jun-Oct)	15/87 (17.2)	6/12(50.0)	8/24 (33.3)	29/123 (23.6)
Winter (Nov- Feb)	1/6 (16.7)	15/29 (51.7)	2/8 (25.0)	18/43 (41.9)
	28/150 (18.7)	21/41 (51.2)	12/47 (25.5)	61/238 (25.6)

* Asymptomatic = clinically healthy cats. ** FIP-suspected = cats with clinical signs and developing effusion in their body cavity. *** Unknown status = sick cats with various clinical signs, e.g., fever, diarrhea, vomiting, anorexia, coughing, and chronic diseases.

The genotype identification of FCoVs by the S gene-specific RT-PCR assay was performed. Our result revealed that all FCoV-positive samples (n = 61) were identified as FCoV type I (Supplementary Table S3). Of 61 FCoV-positive samples, 24 representative samples were selected based on the date, location, and clinical status of the animal. Then, the samples were subjected to genetic characterization by whole genome and S gene sequencing. In this study, 3 whole genome sequences (CU27724, CU27528, and CUFIP486) were obtained. In addition, completed and partial S gene sequences of representative FCoVs (n = 21) were obtained (Table 2). For pairwise comparison, Thai-FCoV (CU27724) had high nucleotide and amino acid identities to FCoV type I from China (strain SD, 91.6% nt identities, 95.4% aa identities), and the Netherlands (strain UU10, 91.2% nt identities, 94.8% aa identities). However, Thai-FCoV has low identities with FCoV type II from the USA (Vaccine strain DF2, 83.2% nt identities, 86.1% aa identities). Moreover, Thai-FCoV possesses 74.5–75.5% (nt identities) and 68.0–74.0% (aa identities) with the other reference Alphacoronaviruses (Table 3). For S gene analysis, identities within the Thai-FCoVs ranged from 83.6–86.9% nucleotide identities and 87.5–92.1% amino acid identities. While Thai-FCoV had high identities with FCoV type I from China (HLJ/HRB/2016/10, 87.0% nt, 92.4% aa), it possessed a remarkably low identity of 30.9% (nt) and 22.8% (aa) with the FCoV type II, vaccine strain (DF2) (Table 3).

**Table 2:** Description of Thai-FCoVs characterized in this study.

Virus ID	Location	Date	Age	Sex	Clinical Signs	Source	Genotype	Sequencing	Size (bp)	GenBank Accession #
CU26828	Nonthaburi	21-Mar	N/A	F	Asymptomatic	Rectal swab	FCoV-I	S^d^	1899	PP901879
CU26882	Bangkok	21-Mar	2M	M	Unknown status	Rectal swab	FCoV-I	S^d^	1899	PP901880
CU26956	Nonthaburi	21-Apr	N/A	FS	Unknown status	Rectal swab	FCoV-I	S^c^	4,407	PP901870
CU27009	Nonthaburi	21-Apr	4 M	M	Asymptomatic	Rectal swab	FCoV-I	S^c^	4,404	PP901871
CU27468	Bangkok	21-Jun	>4 M	M	Unknown status	Rectal swab	FCoV-I	S^c^	4,404	PP901872
CU27529	Bangkok	21-Jul	3 M	M	Asymptomatic	Rectal swab	FCoV-I	S^c^	4,401	PP901873
CU27541	Samut Prakan	21-Aug	7Y	M	Asymptomatic	Rectal swab	FCoV-I	S^d^	1899	PP901881
CU27697	Bangkok	21-Aug	2 M	F	Unknown status	Rectal swab	FCoV-I	S^c^	4,407	PP901874
CU27717	Nonthaburi	21-Aug	N/A	FS	Unknown status	Rectal swab	FCoV-I	S^c^	4,404	PP901875
CU27740	Bangkok	21-Aug	6 M	M	Unknown status	Rectal swab	FCoV-I	S^c^	4,407	PP901876
CU27788	Bangkok	21-Sep	3 Y	F	Unknown status	Rectal swab	FCoV-I	S^c^	4,410	PP901877
CUFIP601	Bangkok	21-Feb	N/A	N/A	FIP-suspected	Abdominal fluid	FCoV-I	S^d^	1899	PP901882
CUFIP2175	Bangkok	21-Aug	N/A	N/A	FIP-suspected	Abdominal fluid	FCoV-I	S^d^	1899	PP901883
CUFIP2921	Bangkok	21-Oct	N/A	N/A	FIP-suspected	Abdominal fluid	FCoV-I	S^d^	1899	PP901884
CUFIP2922	Bangkok	21-Oct	6 M	F	FIP-suspected	Abdominal fluid	FCoV-I	S^c^	4,407	PP901878
CUFIP2960	Bangkok	21-Oct	5 M	F	FIP-suspected	Abdominal fluid	FCoV-I	S^d^	1899	PP901885
CUFIP3011	Bangkok	21-Nov	8 M	M	FIP-suspected	Abdominal fluid	FCoV-I	S^d^	576	PP901886
CUFIP3031	Bangkok	21-Nov	5 M	M	FIP-suspected	Abdominal fluid	FCoV-I	S^d^	576	PP901887
CUFlP3161	Bangkok	21-Nov	N/A	F	FIP-suspected	Abdominal fluid	FCoV-I	S^d^	576	PP901888
CUFIP3175	Bangkok	21-Nov	4 M	M	FIP-suspected	Abdominal fluid	FCoV-I	S^d^	576	PP901889
CUFIP3280	Nonthaburi	21-Nov	N/A	M	FIP-suspected	Abdominal fluid	FCoV-I	S^d^	576	PP901890
CU27528	Bangkok	21-Jul	3 M	M	Asymptomatic	Rectal swab	FCoV-I	WGS^a^	28,717	PP908788
CU27724	Nonthaburi	21-Aug	N/A	M	Asymptomatic	Rectal swab	FCoV-I	WGS^a^	28,653	PP908789
CUFIP486	Bangkok	21-Feb	N/A	F	FIP-suspected	Abdominal fluid	FCoV-I	WGS^b^	28,711	PP908790

WGS^a^ includes ORF1ab, S, ORF3abc, E, M, N, ORF7ab. WGS^b^ includes partial ORF1ab, partial S, ORF3abc, E, M, N, ORF7ab. S^c^ represents the completed S gene. S^d^ represents the partial S gene. N/A: not available.

**Table 3:** Pairwise comparison of whole genome nucleotide and amino acid sequences of Thai-FCoV (CU27724) with reference alpha coronaviruses.

Virus	Accession	Genotype	Host	Country	Year	% nt identity (% aa identities)										
						WGS	ORF1ab	S	3a	3b	3c	E	M	N	7a	7b
**CU27724** *	**This study**	**FCoV-I**	**Feline**	**Thailand**	**2021**											
CU27528	This study	FCoV-I	Feline	Thailand	2021	90.6 (94.8)	91.6 (95.9)	83.7 (89.6)	94.1 (95.6)	93.3 (86.9)	96.8 (96.4)	94.1 (98.8)	90.9 (92.0)	89.3 (92.9)	93.4 (99.0)	92.0 (91.8)
CUFIP486	This study	FCoV-I	Feline	Thailand	2021	(−)	(−)	(−)	92.5 (95.6)	92.5 (88.2)	95.9 (95.4)	95.4 (95.0)	90.3 (92.9)	91.3 (93.7)	93.8 (96.0)	92.9 (93.4)
CU26956	This study	FCoV-I	Feline	Thailand	2021	(−)	(−)	86.1 (90.9)	(−)	(−)	(−)	(−)	(−)	(−)	(−)	(−)
CU27009	This study	FCoV-I	Feline	Thailand	2021	(−)	(−)	83.8 (88.6)	(−)	(−)	(−)	(−)	(−)	(−)	(−)	(−)
CU27468	This study	FCoV-I	Feline	Thailand	2021	(−)	(−)	86.9 (92.1)	(−)	(−)	(−)	(−)	(−)	(−)	(−)	(−)
CU27529	This study	FCoV-I	Feline	Thailand	2021	(−)	(−)	84.1 (90.4)	(−)	(−)	(−)	(−)	(−)	(−)	(−)	(−)
CU27697	This study	FCoV-I	Feline	Thailand	2021	(−)	(−)	86.3 (90.6)	(−)	(−)	(−)	(−)	(−)	(−)	(−)	(−)
CU27717	This study	FCoV-I	Feline	Thailand	2021	(−)	(−)	83.8 (87.5)	(−)	(−)	(−)	(−)	(−)	(−)	(−)	(−)
CU27740	This study	FCoV-I	Feline	Thailand	2021	(−)	(−)	83.6 (89.2)	(−)	(−)	(−)	(−)	(−)	(−)	(−)	(−)
CU27788	This study	FCoV-I	Feline	Thailand	2021	(−)	(−)	82.4 (88.3)	(−)	(−)	(−)	(−)	(−)	(−)	(−)	(−)
CUFIP2922	This study	FCoV-I	Feline	Thailand	2021	(−)	(−)	85.5 (89.3)	(−)	(−)	(−)	(−)	(−)	(−)	(−)	(−)
Type I reference strains																
ZJU1709	MT239440	FCoV-I	Feline	China	2017	91.5 (94.9)	92.7 (96.6)	84.2 (88.2)	93.6 (94.0)	95.2 (90.3)	95.2 (94.5)	94.1 (98.8)	90.1 (91.6)	91.5 (93.4)	95.2 (97.0)	90.8 (92.3)
HLJ/HRB/2016/13	KY566211	FCoV-I	Feline	China	2016	91.0 (95.0)	91.7 (96.0)	86.4 (92.1)	92.0 (89.3)	93.8 (92.3)	96.3 (96.8)	92.3 (96.3)	88.4 (90.7)	90.9 (92.0)	95.2 (96.0)	90.4 (90.2)
HLJ/HRB/2016/11	KY566210	FCoV-I	Feline	China	2016	91.0 (95.0)	91.7 (96.0)	86.7 (92.2)	93.0 (94.0)	91.8 (88.2)	96.3 (96.8)	92.3 (96.3)	88.4 (90.7)	90.9 (92.0)	95.2 (96.0)	90.4 (90.2)
HLJ/HRB/2016/10	KY566209	FCoV-I	Feline	China	2016	91.1 (95.0)	91.7 (96.0)	87.0 (92.4)	92.0 (89.3)	93.8 (92.3)	96.3 (96.8)	92.3 (96.3)	88.4 (90.7)	91.3 (92.9)	95.2 (96.0)	90.4 (90.2)
HLJ/DQ/2016/01	KY292377	FCoV-I	Feline	China	2016	91.5 (90.0)	92.6 (96.5)	84.0 (90.0)	92.0 (94.0)	90.5 (86.1)	96.3 (95.9)	94.1 (98.8)	89.0 (92.1)	93.0 (94.3)	95.2 (96.0)	92.7 (92.9)
SD	MW030110	FCoV-I	Feline	China	2018	91.6 (95.4)	92.6 (96.5)	85.3 (91.3)	92.0 (94.0)	89.0 (83.6)	96.3(95.0)	92.3 (95.0)	92.8 (94.5)	90.8 (93.1)	94.5 (98.0)	91.0 (91.3)
QS	MW030108	FCoV-I	Feline	China	2018	91.0 (94.9)	92.4 (96.1)	87.5 (90.9)	91.8 (96.4)	81.8 (86.6)	95.4 (94.5)	95.0 (99.9)	90.6 (92.4)	91.0 (92.2)	95.2 (97.0)	92.0 (90.2)
UU9	FJ938062	FCoV-I	Feline	Netherlands	2007	91.2 (94.8)	92.3 (96.4)	84.0 (88.4)	93.6 (92.4)	90.5 (88.2)	94.8 (94.0)	94.5 (97.5)	92.2 (92.9)	92.4 (94.0)	95.6 (99.0)	91.2 (90.7)
UU22	GU553361	FCoV-I	Feline	Netherlands	2007	90.5 (94.4)	91.6 (95.8)	83.3 (87.6)	93.0 (90.9)	90.5 (90.3)	95.9 (95.9)	93.7 (98.8)	90.2 (93.7)	91.3 (94.5)	94.5 (97.0)	92.3 (91.2)
UU2	FJ938060	FCoV-I	Feline	Netherlands	2007	90.3 (94.3)	91.5 (95.5)	83.7 (89.4)	92.5 (94.0)	91.2 (90.3)	95.7 (95.4)	91.9 (95.0)	89.5 (93.2)	88.5 (92.5)	94.9 (97.1)	91.2 (90.2)
UU88	KF530123	FCoV-I	Feline	Netherlands	2007	90.5 (94.6)	91.3 (95.8)	85.3 (90.6)	90.8 (92.5)	89.2 (86.1)	96.3 (96.4)	94.5 (97.5)	90.7 (94.5)	90.7 (93.7)	92.7 (93.1)	90.4 (89.5)
UU19	HQ392470	FCoV-I	Feline	Netherlands	2007	90.4 (94.2)	91.6 (95.6)	82.9 (89.2)	92.0 (92.5)	91.3 (88.2)	96.0 (96.4)	94.5 (98.8)	89.3 (91.6)	91.3 (92.3)	93.4 (95.9)	91.0 (87.9)
UU17	HQ012367	FCoV-I	Feline	Netherlands	2007	90.0 (93.6)	91.4 (95.4)	81.7 (86.9)	90.4 (87.7)	91.3 (88.2)	94.9 (94.9)	92.8 (96.3)	88.9 (91.2)	91.0 (92.0)	93.4 (94.7)	89.3 (85.7)
UU10	FJ938059	FCoV-I	Feline	Netherlands	2007	91.2 (94.8)	92.4 (96.2)	84.0 (88.7)	93.1 (94.0)	93.8 (88.0)	96.5 (95.9)	93.7 (96.3)	90.4 (92.2)	92.4 (94.4)	94.2 (99.0)	92.6 (93.4)
UG-FH8	KX722529	FCoV-I	Feline	Belgium	2015	90.6 (94.8)	91.9 (96.3)	82.7 (88.8)	93.6 (95.6)	91.9 (86.1)	95.4 (95.9)	95.9 (98.8)	90.1 (92.1)	91.7 (94.5)	95.3 (97.0)	90.5 (89.1)
Cat2 day21	KU215422	FCoV-I	Feline	Belgium	2013	90.3 (94.3)	91.5 (95.5)	83.7 (89.4)	92.5 (94.0)	91.2 (90.3)	95.7 (95.4)	91.9 (95.0)	89.3 (92.8)	88.4 (94.5)	94.9 (96.0)	91.2(90.2)
Cat_1_Karlslunde	KX722530	FCoV-I	Feline	Denmark	2015	90.1 (94.2)	90.9 (95.4)	84.9 (90.9)	92.5 (94.0)	91.2 (90.3)	95.7 (95.4)	91.9 (95.0)	89.3 (92.8)	88.4 (92.5)	94.9 (96.9)	86.9 (81.1)
Felix	MG893511	FCoV-I	Feline	Germany	2021	90.4 (94.0)	92.0 (95.7)	82.0 (87.9)	88.7 (87.7)	84.6 (85.8)	96.2 (95.4)	91.9 (96.3)	89.4 (90.8)	90.7 (92.5)	93.4 (97.0)	88.9 (89.6)
SB-22	MH817484	FCoV-I	Feline	Brazil	2015	90.0 (93.9)	91.5 (95.5)	81.6 (87.9)	89.8 (89.2)	87.7 (86.1)	95.1 (95.0)	93.2 (97.5)	90.0 (92.4)	89.5 (92.2)	91.6 (94.9)	91.6 (90.7)
RM	FJ938051	FCoV-I	Feline	USA	2002	89.9 (94.1)	91.2 (95.5)	83 (89.3)	90.4 (94.0)	84.9 (74.9)	93.5 (92.1)	94.1 (96.2)	88.9 (93.2)	89.8 (91.9)	93.4 (93.9)	89.5 (92.3)
Black	EU186072	FCoV-I	Feline	USA	1970	91.2 (94.2)	92.8 (96.0)	82.8 (87.5)	90.9 (87.7)	87.7 (82.9)	93.5 (90.3)	92.3 (91.1)	90.5 (92.4)	89.8 (92.0)	92.7 (96.0)	88.9 (88.5)
C1Je	DQ848678	FCoV-I	Feline	UK	2006	87.2 (92.0)	88 (93.6)	79.3 (85.5)	89.7 (94.0)	88.4 (84.0)	95.8 (95.0)	92.8 (97.5)	89.7 (91.2)	89.2 (92.0)	95.5 (97.0)	89.5 (85.2)
80F	KP143511	FCoV-I	Feline	UK	2013	88.6 (93.6)	89.9 (95.3)	79.3 (87.3)	90.9 (89.3)	89.2 (88.2)	94.9 (94.0)	93.7 (98.8)	90.9 (92.4)	90.5 (92.6)	93.1 (96.1)	89.3 (87.4)
67C	KP143510	FCoV-I	Feline	UK	2013	88.5 (93.5)	89.9 (95.2)	79.2 (86.9)	89.9 (89.3)	86.3 (84.0)	95.4 (93.5)	94.5 (97.5)	89.0 (93.3)	89.0 (92.6)	94.1 (93.9)	89.3 (87.4)
26 M	KP143512	FCoV-I	Feline	UK	2013	88.5 (93.4)	89.9 (95.2)	79.1 (86.4)	89.9 (89.3)	86.3 (84.0)	95.3 (93.4)	94.5 (97.5)	89.1 (93.3)	88.8 (92.3)	93.8 (94.9)	89.3 (87.4)
Type II reference strains																
FCoV/NTU156/P/2007	GQ152141	FCoV-II	Feline	Taiwan	2007	82.8 (85.8)	89.6 (94.6)	30.9 (21.9)	64.2 (65.8)	46.6 (34.3)	81.8 (973.8)	74.2 (73.6)	81.8 (86.4)	91.3 (93.9)	97.0 (99.0)	90.3 (86.9)
DF2-R3i	JQ408980	FCoV-II	Feline	Hungary	2007	83.2 (86.2)	89.3 (94.4)	30.9 (22.5)	71.0 (73.4)	48.9 (37.8)	90.0 (86.1)	94.1 (96.3)	90.0 (92.4)	90.0 (91.7)	93.4 (96.0)	89.3 (85.7)
WSU79-1683	JN634064	FCoV-II	Feline	USA	2005	81.4 (85.1)	88.4 (94.2)	29.8 (21.4)	64.3 (65.8)	35.1 (24.4)	85.9 (83.0)	69.7 (75.2)	90.1 (92.8)	90.1 (92.0)	93.4 (94.9)	89.3 (85.3)
79–1,146 (vaccine)	AY994055	FCoV-II	Feline	USA	2005	83 (86.0)	89.5 (94.7)	30.7 (22.9)	65.9 (65.8)	43.1 (19.4)	91.7 (93.1)	94.1 (96.3)	79.6 (82.4)	90.7 (93.4)	93.8 (96.0)	84.7 (86.3)
DF2 (vaccine)	DQ286389	FCoV-II	Feline	USA	2005	83.2 (86.1)	89.3 (94.7)	30.9 (22.8)	71.0 (66.4)	NA	87.2 (83.8)	94.1 (96.3)	90.0 (92.4)	90.0 (91.7)	93.4 (96.0)	NA
Alphacoronavirus 1 reference strain																
1_71	JQ404409	CCoV-II	Canine	Germany	1971	75.5 (69.5)	81.1 (88.1)	30.4 (22.6)	63.9 (62.2)	54.0 (27.1)	87.8 (85.6)	75.4 (79.5)	76.3 (81.0)	72.7 (75.1)	84.6 (80.0)	62.3 (50.7)
A76	JN856008	CCoV-II	Canine	USA	1976	75.4 (69.2)	81.1 (88.1)	33.9 (31.5)	64.3 (61.6)	NA	87.1 (85.6)	76.1 (73.6)	77.0 (80.9)	73.5 (74.8)	82.5 (80.4)	64.8 (55.4)
HLJ-071	KY063616	FCoV-II	Canine	China	2016	75.1 (68.4)	81.2 (88.4)	30.2 (22.1)	64.2 (65.8)	44.1 (26.9)	86.5 (85.1)	73.5 (72.0)	75.9 (80.0)	73.3 (75.8)	81.6 (80.4)	65.8 (56.2)
2020/15	MT906864	CCoV-II	Canine	UK	2020	75.5 (68.0)	81.1 (88.1)	34.8 (32.8)	65.0 (65.8)	46.4 (30.7)	87.8 (85.1)	74.8 (72.9)	76.5 (81.3)	73.2 (74.8)	82.9 (77.9)	63.8 (55.4)
2020/7	MT906865	CCoV-II	Canine	UK	2020	75.2 (69.5)	81.4 (88.4)	27.6 (20.8)	65.1 (63.7)	44.8 (23.0)	87.4 (85.1)	73.7 (72.0)	77.0 (81.4)	73.9 (76.5)	83.4 (80.4)	61.9 (51.5)
23/03	KP849472	CCoV-I	Canine	Italy	2003	75 (74.0)	81.1 (87.8)	66.1 (71.2)	69.5 (65.8)	46.1 (34.3)	89.0 (84.6)	77.4 (82.7)	77.9 (84.3)	74.4 (73.3)	86.7 (80.4)	68.4 (54.4)
HuPn	MW591993	CCoV-II	Human	Malaysia	2018	75.1 (68.0)	81.0 (87.7)	29.6 (63.7)	65.8 (63.7)	47.5 (30.7)	87.8 (86.1)	75.7 (72.0)	75.5 (80.0)	73.3 (74.5)	81.2 (86.2)	63.8 (53.9)
Z19	MZ420153	CCoV-II	Human	Haiti	2017	74.9 (68.0)	80.8 (87.6)	29.6 (63.7)	65.8 (63.7)	47.5 (30.7)	87.8 (86.1)	72.9 (73.6)	77.6 (81.5)	73.9 (75.8)	78.9 (77.9)	64.7 (55.4)
Virulent purdue	DQ811789	NA	Swine	China	2004	74.5 (68.1)	80.4 (87.1)	30.6 (22.0)	63.5 (61.6)	NA	86.3 (84.6)	75.5 (73.6)	75.8 (80.5)	72.0 (73.1)	77.5 (78.3)	NA

The Thai-FCoV (CU27724) is used for comparision with other CoVs.

**Figure 1:**
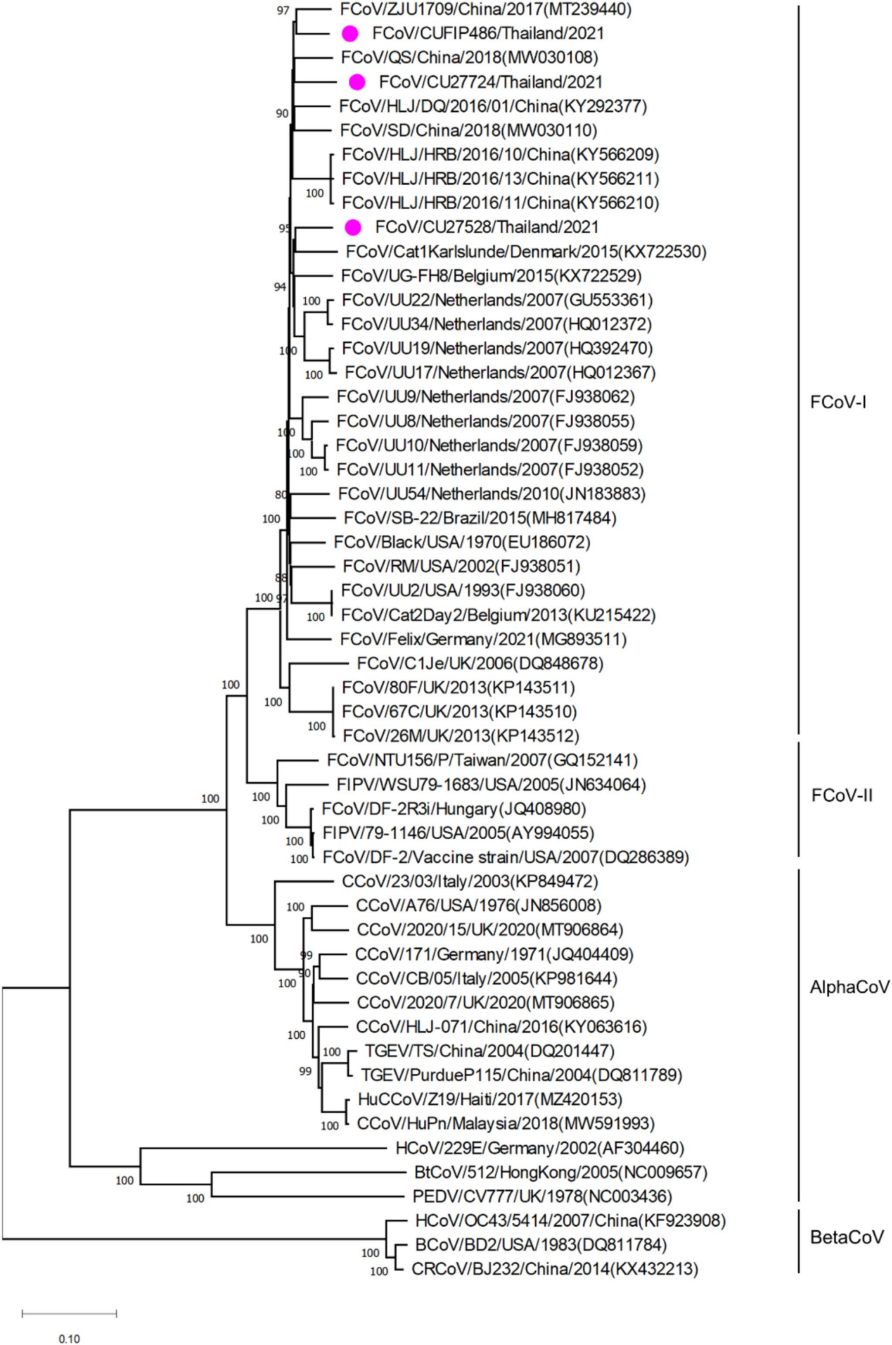
Phylogenetic tree based on whole genome of Thai-FCoVs and reference alphacoronaviruses and betacoronaviruses. The rooted phylogenetic tree was constructed by using MEGA v.7.0 with a neighbor-joining algorithm with kimura-2 parameter model and bootstrap analysis of 1,000 replications. The bootstrap values are displayed next to the nodes. Pink circles indicate the whole genome sequence of Thai-FCoVs.

**Figure 2:**
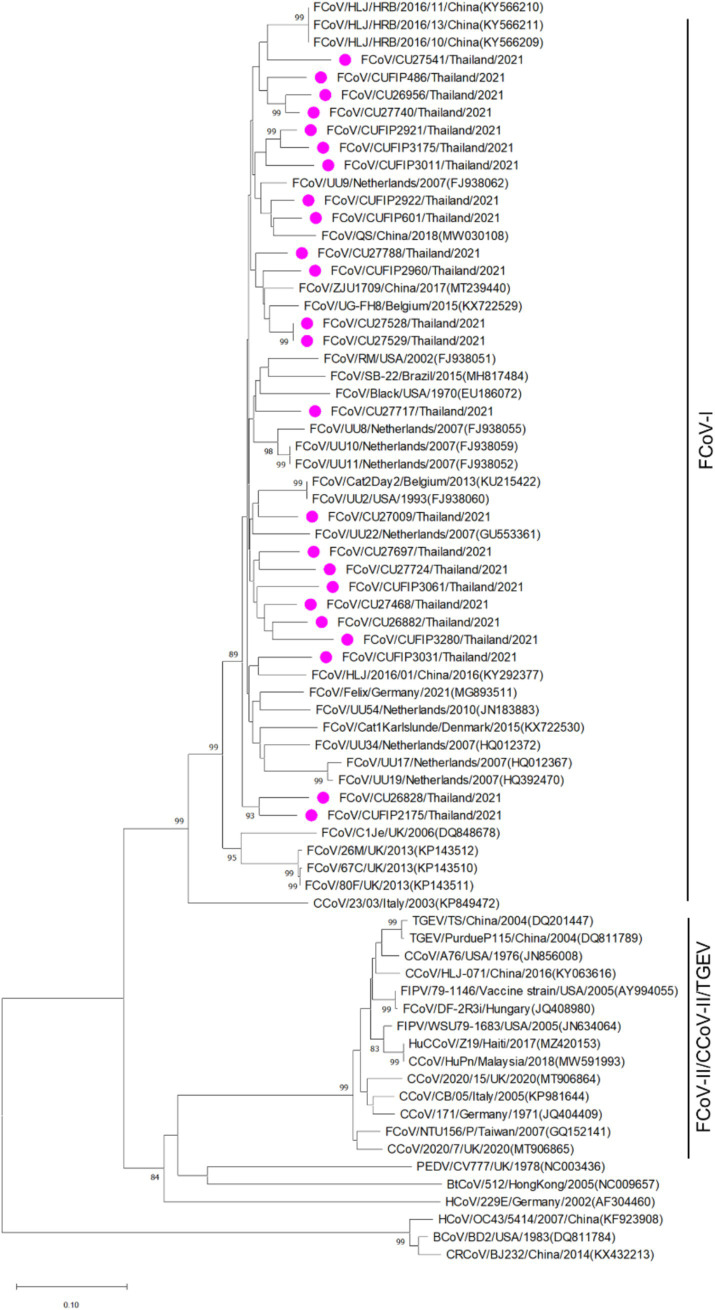
Phylogenetic tree based on the partial S gene of Thai-FCoVs and reference alphacoronaviruses and betacoronaviruses. The rooted phylogenetic tree was constructed by using MEGA v.7.0 with a neighbor-joining algorithm with kimura-2 parameter model and bootstrap analysis of 1,000 replications. The bootstrap values are displayed next to the nodes. Pink circles indicate the partial S gene sequences of Thai-FCoVs.

**Figure 3:**
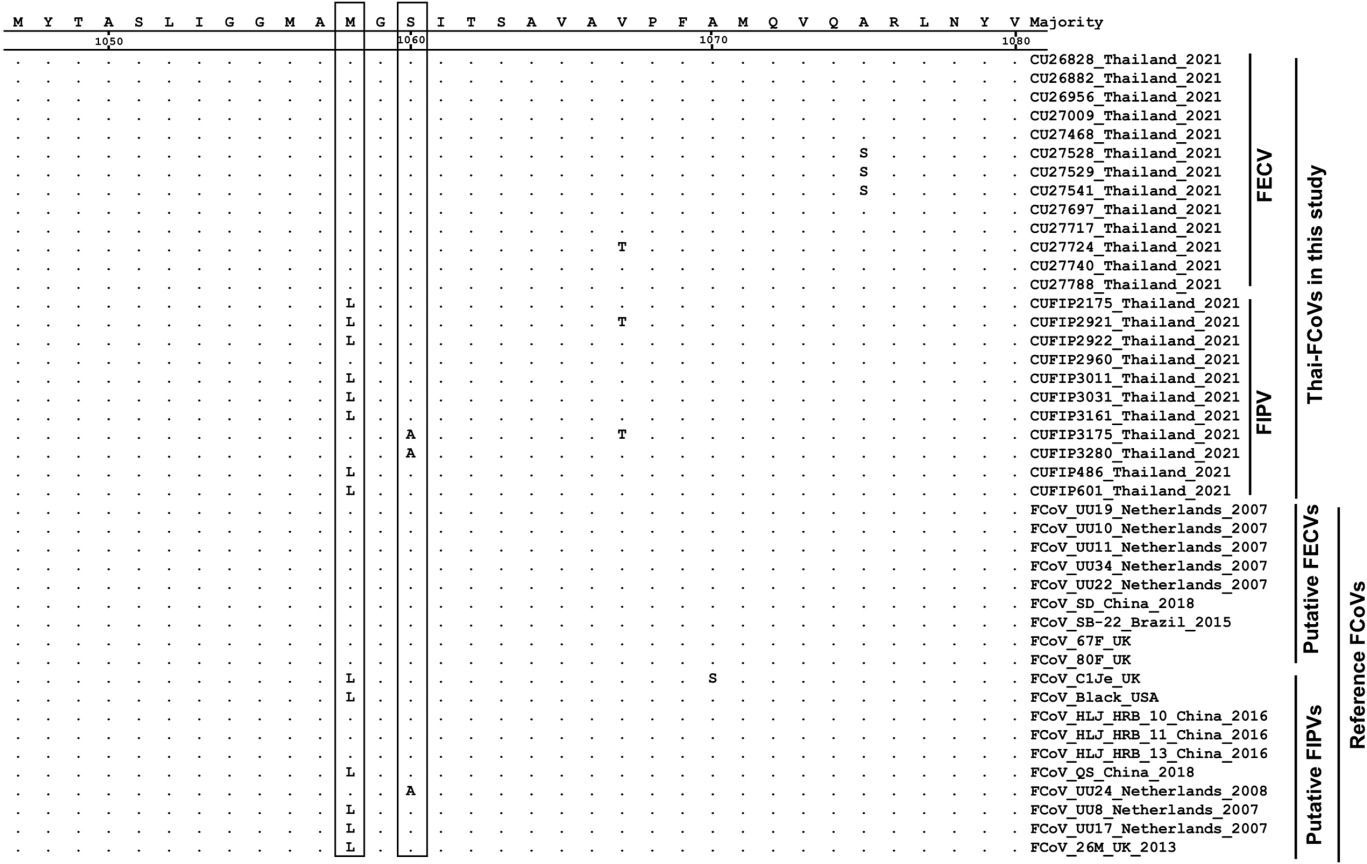
Alignment of amino acids of S gene of Thai-FCoVs with reference FCoVs. The boxes indicate the amino acid substitutions suggesting hotspots for differentiation between two pathotypes.

The phylogenetic tree of whole genome sequences of Thai-FCoVs (CU27724, CU27528, CUFIP486) showed that Thai-FCoVs belonged to FCoV type I. It is noted that the CU27724 and CUFIP486 were closely related to Chinese FCoV (QS and ZJU1709 strain), and the CU27528 was closely related to FCoV from Denmark (Karlslunde strain) (Figure 1). Phylogenetic analysis of the S gene showed that Thai-FCoVs (n = 24) were clustered with FCoV type I (Figure 2). Similar findings were observed in the phylogenetic analyses of each gene (ORF1ab, E, M, N, 7ab) (Supplementary Figures S1–S4). Based on the genetic analysis of the S gene, we analyzed two predicted proteolytic cleavage sites, S1/S2, and S2 regions (8, 9). For the S1/S2 region (R-R-S-R/A-R-S), all Thai-FCoVs contained R-R-S-R/A-R-S, consistent with the reference FCoV type I. In the S2 subunit region (K-R-S), all Thai-FCoVs contained K-R-S, which were identical to reference FCoV type I. It should be noted that our findings suggested that the amino acid residues in the predicted proteolytic cleavage sites are not associated with the transition between two pathotypes, and further analysis is needed. In this study, the putative fusion peptides (positions 1,058 and 1,060) were analyzed as in the previous study (7). All Thai-FCoVs obtained from the rectal swabs from asymptomatic cats or cats with unknown status (*n* = 13), also known as Feline Enteric Coronaviruses (FECV), exhibited a conserved methionine at amino acid position 1,058 (M1058). While 8 out of 11 Thai-FCoVs, obtained from the abdominal fluid of cats suspected to have Feline Infectious Peritonitis (FIPV), showed an amino acid substitution to leucine at position (1,058 L). The association of FIP-suspected cases (FIPV) and genetic mutation at M1058L was statistically significant (*p* < 0.001). To expand this observation, we further analyzed 266 FCoV nucleotide sequences available in the GenBank database. The association of FIP-suspected cases (FIPV) and genetic mutation at M1058L (98 out of 119) was observed, with statistical significance (*p* < 0.001). It is noted that most Thai-FIPVs (except CU3175 and CU3280) and FIPVs in the GenBank database contained S1060 but were not statistically significant (Figure 3; Tables 4, 5).

**Table 4:** Genetic analysis of S gene of Thai-FCoV with reference FCoV-I sequences.

Virus	Country	Year	Putative Pathotypes	S gene (4,404 nt)				
				160–162 insertion	Predicted proteolytic cleavage sites		Fusion peptide	
					S1/S2 (795–800)	S2’ (988–990)		
					R-R-A/S-R-R-S	KRS	M1058 L	S1060A
Reference FCoV type I								
HLJ/HRB/2016/13	China	2016	FECV	HLT	RRSRRS	KRS	M	S
HLJ/HRB/2016/11	China	2016	FECV	HLT	RRSRRS	KRS	M	S
HLJ/HRB/2016/10	China	2016	FECV	HLT	RRSRRS	KRS	M	S
HLJ/DQ/2016/01	China	2016	FECV	-	RRSRRS	KRS	M	S
SD	China	2018	FECV	HLT	RRSRRS	KRS	M	S
UU9	Netherlands	2007	FECV	-	RRSRRL	KRS	M	S
UU22	Netherlands	2007	FECV	-	RRSRRS	RRS	M	S
UU2	Netherlands	2007	FECV	-	RRSRRS	KRS	M	S
UU19	Netherlands	2007	FECV	-	RRSRRS	TRS	M	S
UU10	Netherlands	2007	FECV	-	KRSRRS	KRS	M	S
UG-FH8	Belgium	2015	FECV	-	KRLRRS	KRS	M	S
SB-22	Brazil	2015	FECV	-	RRSRRS	KRS	M	S
RM	USA	2002	FECV	-	RRSRRS	KRS	M	S
Felix	Germany	2021	FECV	-	RRSRRE	KRS	M	S
Cat2_day21	Belgium	2013	FECV	-	RRSRRS	RRS	M	S
80F	UK	2013	FECV	-	RRARRS	KRS	M	S
67C	UK	2013	FECV	-	RRARRS	KRS	M	S
ZJU1709	China	2017	FIPV	-	RRSRRS	KRS	L	S
QS	China	2018	FIPV	HLS	RRSRTS	KRS	L	S
UU17	Netherlands	2007	FIPV	-	RRSRRS	VRS	L	S
Black	USA	1970	FIPV	-	KRSRRP	VRS	L	S
Cat_1_Karlslunde	Denmark	2015	FIPV	-	RRSRGP	KRS	L	S
C1Je	UK	2006	FIPV	-	RQSRRS	KRS	L	S
27 M	UK	2013	FIPV	-	RGARRS	KRS	L	S
This study								
CU27724	Thailand	2021	FECV	HTS	RRSRRS	KRS	M	S
CU27528	Thailand	2021	FECV	-	RRSRRS	KRS	M	S
CU26956	Thailand	2021	FECV	HLS	RRSRRS	KRS	M	S
CU27009	Thailand	2021	FECV	-	KRSRRS	RRS	M	S
CU27468	Thailand	2021	FECV	HLS	KRSRRS	KRS	M	S
CU27529	Thailand	2021	FECV	-	RRSRRS	KRS	M	S
CU27697	Thailand	2021	FECV	HLS	RRSRRS	KRS	M	S
CU27717	Thailand	2021	FECV	-	KRSRRS	KRS	M	S
CU27740	Thailand	2021	FECV	-	RRSRRS	KRS	M	S
CU27788	Thailand	2021	FECV	-	RRARRS	KRS	M	S
CU26828	Thailand	2021	FECV	n/a	n/a	KRS	M	S
CU26882	Thailand	2021	FECV	n/a	n/a	RRS	M	S
CU27541	Thailand	2021	FECV	n/a	n/a	KRS	M	S
CUFIP2922	Thailand	2021	FIPV	HLT	KGARRS	KRS	L	S
CUFIP2960	Thailand	2021	FIPV	n/a	n/a	KRS	M	S
CUFIP2921	Thailand	2021	FIPV	n/a	n/a	KRS	L	S
CUFIP2175	Thailand	2021	FIPV	n/a	n/a	KRS	L	S
CUFIP486	Thailand	2021	FIPV	n/a	n/a	KRS	L	S
CUFIP601	Thailand	2021	FIPV	n/a	n/a	KRS	L	S
CUFIP3011	Thailand	2021	FIPV	n/a	n/a	n/a	L	S
CUFIP3031	Thailand	2021	FIPV	n/a	n/a	n/a	L	S
CUFlP3161	Thailand	2021	FIPV	n/a	n/a	n/a	L	S
CUFIP3175	Thailand	2021	FIPV	n/a	n/a	n/a	M	A
CUFIP3280	Thailand	2021	FIPV	n/a	n/a	n/a	M	A

*n/a = not available.

**Table 5:** Analysis of the association of FIP-related clinical presentations and amino acid substitution at positions 1,058 and 1,060 of fusion protein (S protein).

	Amino acid substitution at fusion protein (S protein)					
This study						
Thai FCoV (*n* = 24)						
	M1058^a^	1,058 L^a^		S1060^b^	1060A^b^	
FIP-suspected cats	3	8		9	2	
Cats with asymptomatic and unknown status	13	0		13	0	
			*P* < 0.001*			*p* = 0.108
GenBank database						
Available FCoV (*n* = 266)						
	M1058	1,058 L		S1060	1060A	
FIP-suspected cats	21	98		115	4	
Cats with asymptomatic and unknown status	145	2		145	2	
			*P* < 0.001*			*p* = 0.275

a Amino acid substitution at position 1,058 (M1058L).

b Amino acid substitution at position 1,060 (S1060A).

*Statistical significance, *p*-value < 0.01.

**Figure 4:**
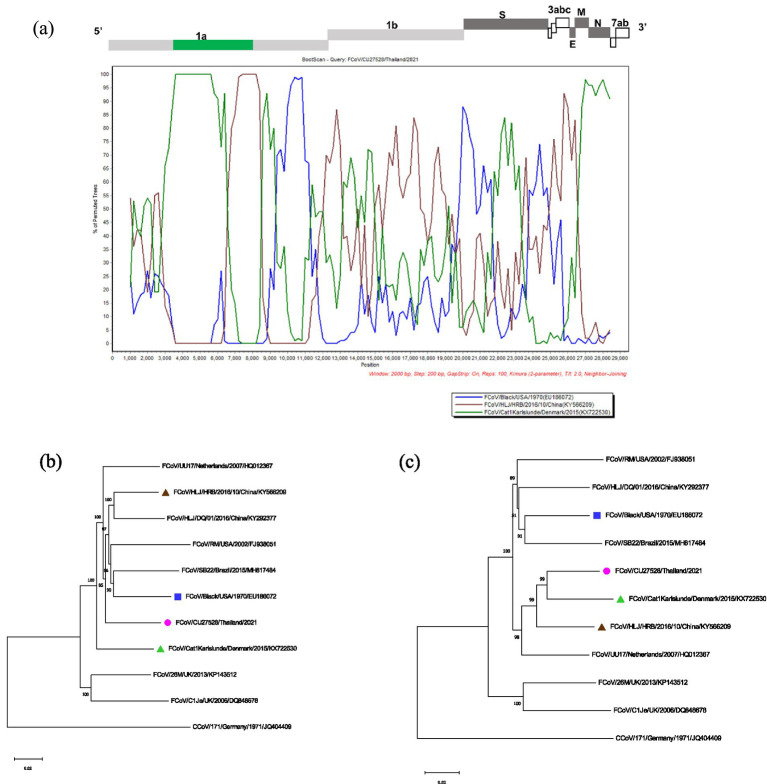
Results of recombination analysis of CU27528. **(A)** Bootscan analysis of Thai-FCoV indicates the recombinant CU27528. **(B)** The phylogenetic tree was constructed based on the region derived from the major parent **(C)** The phylogenetic tree was constructed based on the region derived from the minor parent. The pink circle in phylogenetic analysis indicates the Thai-FCoV strain, and the blue square and green triangle indicate the potential major parent strain and minor parent strain, respectively.

**Figure 5:**
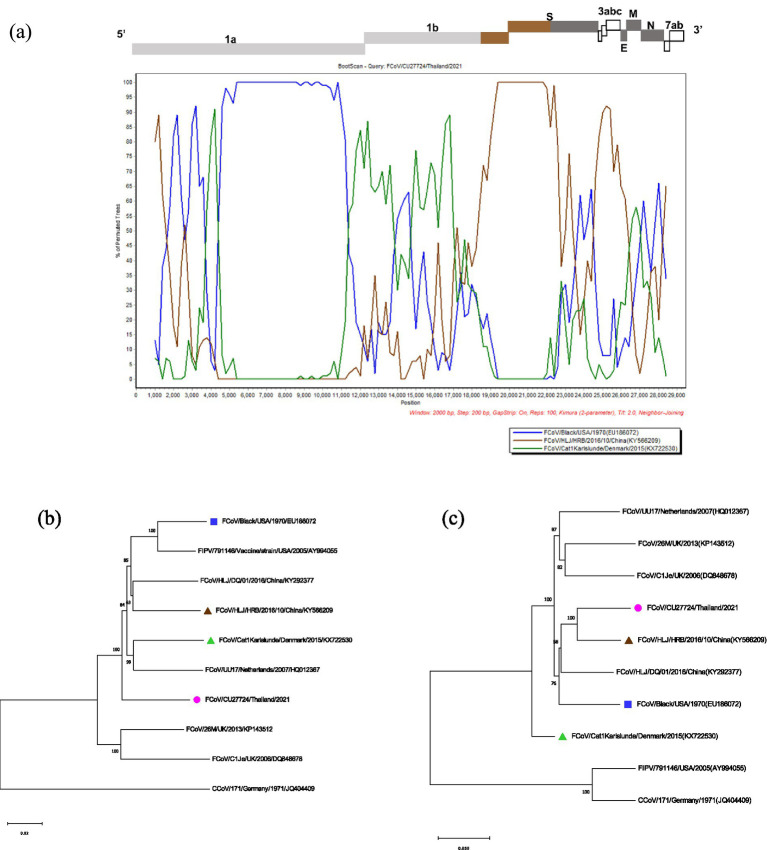
Results of recombination analysis of CU27724. **(A)** Bootscan analysis of Thai-FCoV indicates the recombinant CU27724. **(B)** The phylogenetic tree was constructed based on the region derived from the major parent **(C)** The phylogenetic tree was constructed based on the region derived from the minor parent. The pink circle in phylogenetic analysis indicates the Thai-FCoV strain, and the blue square and brown triangle indicate the potential major parent strain and minor parent strain, respectively.

The potential recombination events were analyzed using the RDP5 and confirmed by the bootscan analysis of Simplot software (21, 22). The whole genome sequences of Thai-FCoVs (CU27528, CU27724) from this study were selected for recombination analysis. For CU27528, the recombination event was found at the ORF1ab gene with significant *p-values* (Figure 4). FCoV/Black/USA/1970 (Blue line) and FCoV/Cat1Karlslunde/Denmark/2015 (Green line) were identified as the major parent and minor parent, respectively. The Simplot results indicated that the majority of the genome of CU27528 is derived from FCoV/Black/USA/1970, except at nucleotide position 4,316–11,068, suggesting acquisition from FCoV, Karlslunde strain. The phylogenetic analysis also supported this recombination pattern (Figure 4). For CU27724, the recombination event was identified at the 3′ end of ORF1b and two-thirds of the S gene, with significant *p-values* (Figure 5). FCoV/Black/USA/1970 (Blue line) and FCoV/HLJ/HRB/10/China/2016 (Brown line) were identified to be the major and minor parent, respectively. The Simplot results showed that CU27724 contains most of its genome from the FCoV Black strain, except at nucleotide position 18,204–23,093, which was acquired from the FCoV Chinese strain. The phylogenetic analysis also supports this recombination pattern (Figure 5).

## Discussion

Feline coronavirus (FCoV) is a significant pathogen in wild and domestic cat populations. FCoV can cause mild enteritis to the fatal disease named feline infectious peritonitis (FIP). In Thailand, there have been only three reports of FCoVs, and information on the whole genome of this virus remains lacking (15–17). To our knowledge, this study is the first report on the whole genome characterization of feline coronavirus (FCoV) from cats in Thailand. Our study provided whole genome sequences and spike gene sequences of Thai-FCoVs and contributed to the expansion of genetic information of FCoVs to the scientific database.

In this study, we found FCoVs in 18.7% of asymptomatic cats, 25.5% of cats with unknown status, and 51.2% of FIP-suspected cats. A 51.2% of FIP-suspected cats tested positive for FCoV, which was higher than the reports from Korea (19.3%), Thailand (46.0%), and Turkey (37.3%) (15, 23, 24), but lower than the report in China (75.7%) (18). It is important to note that the high percentage of FCoV positive in FIP-suspected cats in this study may be due to the bias in sampling for testing FIP-suspected cases and diagnostic services. In this study, we observed that FCoV was more frequently detected in cats younger than 6 months old, consistent with previous studies. For example, studies in China and Japan reported that FCoV was mostly detected in younger cats (14, 18, 25, 26). However, a few reports from Australia, Hungary, and Malaysia suggested that FCoV infection was not related to age (27–29). In contrast, our results suggested that young age may be associated with the higher occurrence of FCoV in cats with statistical significance. Since FCoV was detected in cats of all ages, it is possible that some cats could be chronically infected and become asymptomatic carriers. In this study, FCoV infection in male cats was slightly higher than in female cats but not statistically significant, consistent with other studies (14, 30, 31). In this study, FCoV can be detected year-round and is more frequently found in the winter, suggesting a possible seasonal pattern of FCoV in cats but not statistical significance. However, there are some reports that FCoV was not related to the season (25, 32, 33). These data suggest that the association between age, sex, weather, and susceptibility/resistance to FCoV remains uncertain due to no statistical significance. Our study categorized cats into three groups based on their clinical status: asymptomatic cats, cats with unknown status (cats with other clinical symptoms), and FIP-suspected cats (cats with clinical signs and development of effusion in body cavities). Our results showed that FCoV could be detected in cats regardless of their clinical status, which is in line with previous studies (15, 18). Regardless of their clinical status, the consistent positive rate of FCoVs in cats indicates that asymptomatic cats could serve as reservoirs for susceptible animals. This finding raises concern about the prevention and control of the virus.

FCoVs can be classified into two distinct genotypes, FCoV type I and FCoV type II, based on genetic variations in the S gene (11). Our results showed that all positive samples were identified as FCoV type I, while FCoV type II could not be detected. Our finding aligns with a previous study in Thailand, supporting the predominance of FCoV type I in the country (15). In contrast, FCoV type II is less predominant in the field (34, 35). The phylogenetic tree of the whole genome also showed that Thai-FCoVs belonged to FCoV type I, and were closely related to FCoVs from China and Europe. This suggested that FCoV type I predominantly circulates in domestic cats in Thailand and shared common ancestors with FCoVs from China and Europe.

Previous studies have proposed that mutations in S genes, especially in the proteolytic sites in the S1/S2 subunit and S2 subunit, were responsible for the pathotype switch from FECV to FIPV and the development of FIP (8, 9). We also analyzed the two mutation sites for the pathotype switch. Our result showed that all Thai-FCoVs associated with asymptomatic animals or animals with unknown status (FECV) exhibited a conserved methionine at amino acid position 1,058 (M1058). On the other hand, 82.4% of Thai-FCoVs associated with FIP-suspected cases (FIPV) had a substitution M1058L, which was statistically significant (*p* < 0.001). Moreover, an analysis of 266 FCoV nucleotide sequences in the GenBank database showed similar results, supporting previous studies (8, 9). While this finding supports the potential link between the M1058L mutation and the transition from FECV to FIPV, other studies suggest that the M1058L mutation may be more related to systemic viral spread rather than a direct pathotype switch (36, 37). This highlights the need for further research to clarify the role of this mutation in FCoV pathogenesis.

Recombination serves as the major mechanism for the evolution and genetic diversity of RNA viruses (38). In this study, we analyzed the potential recombination events in Thai-FCoVs. The results showed that Thai-FCoV (CU27528) acquired its backbone from the Classical FCoV type I Black strain (FCoV/Black/USA/1970), and acquired a fragment in the ORF1a gene (position 4,316–11,068) from FCoV/Cat1Karlslunde/Denmark/2015. Moreover, Thai-FCoV (CU27724) is also likely to receive the majority of its genome from the Classical FCoV type I Black strain (FCoV/Black/USA/1970), where the recombination fragment in the 3′ end of ORF1b and one-third of the S gene (position 18,204–23,093) were derived from FCoV/HLJ/HRB/10/China/2016. This analysis revealed that the diverse FCoVs may have been circulating among domestic cats in Bangkok and the vicinity. Different FCoV strains can coexist and cause mixed infections under natural conditions, resulting in recombination. Thai-FCoVs may have originated from the same parental Black strain, which was isolated in the 1970s. However, Thai-FCoVs possessed different potential minor parents from Chinese and Denmark FCoVs, suggesting continuous evolution through recombination among different FCoV strains over time. The identification of FCoV recombination events in Thai-FCoVs from geographically distant strains, such as FCoVs from China, Denmark, and the USA, highlights the global spread of FCoVs in domestic cats. Recombination events can contribute to the emergence of FCoV strains with changes in virulence, transmission, and host range in the future.

In conclusion, we reported the first whole genome sequence of FCoVs in Thailand. This study provided the occurrence of FCoV in domestic cats in Thailand and identified an association between FCoV occurrence and age, sex, and seasonal variations. The predominant type of FCoV circulating in domestic cats in Thailand was FCoV type I. Thai-FCoVs were closely related to FCoVs from China and Europe. Most Thai-FIPVs exhibited amino acid mutation (M1058L), likely responsible for the pathotype switch. Recombination breakpoints were mainly observed in the ORF1ab and S genes of FCoVs. Our findings highlight the importance of routine surveillance and public education in monitoring, preventing, and controlling FCoVs in domestic cats.

## Data Availability

The authors declare that the data supporting the findings of this study can be obtained upon request. The nucleotide sequence data are available in the GenBank database, under accession numbers # PP901870-PP901890 and PP908788-PP908790.

## Footnotes

^1^ https://www.genomedetective.com/app/typingtool/virus

^2^ https://digitalinsights.qiagen.com/products/qiagen-clc-main-workbench/
