# Supplementary material for: Whole genome characterization of feline coronaviruses in Thailand: evidence of genetic recombination and mutation M1058L in pathotype switch
Source: Front Vet Sci. 2025 Feb 14;12:1451967. doi: 10.3389/fvets.2025.1451967 (PMC11869453; doi:10.3389/fvets.2025.1451967)

Supplement materials

Supplement Table

Supplement Table 1. Nucleotide sequences of primers used for FCoV sequencing in this study.

Supplement Table 2. Statistical analysis of the association of FCoV positive rate and demographics and seasonal pattern

Supplement Table 3. Genotype identification of positive Thai-FCoVs (n=61) in this study

Supplement Figures

Supplement Figure 1. Phylogenetic tree based on ORF1ab gene of Thai-FCoVs and reference alphacoronaviruses and betacoronaviruses

Supplement Figure 2. Phylogenetic tree based on ORF3abc gene of Thai-FCoVs and reference alphacoronaviruses and betacoronaviruses

Supplement Figure 3. Phylogenetic tree based on E, M, N gene of Thai-FCoVs and reference alphacoronaviruses and betacoronaviruses

Supplement Figure 4. Phylogenetic tree based on ORF7ab gene of Thai-FCoVs and reference alphacoronaviruses and betacoronavirses

Supplement Table 1. Nucleotide sequences of primers used for FCoV sequencing in this study.

**Primer name**	**Forward (5'-3')**	**Primer name**	**Reward (5'-3')**	**Position**	**Gene**	**Product size**	**Reference**
FCOV 1-2_104_1F	GGAACGGGGTTGAGAGAACG	FCOV 1-2_1756_1R	AATGCACTATCRAGACCTTT	104-1756	ORF1ab (Type I and II)	1652	This study
FCOV 1-2_983_2F	GGTGTWGGTGAYTGGACTGG	FCOV 1-2_2441_2R	CATTRCCTAGCATRATTTTAAC	983-2441	ORF1ab (Type I and II)	1458	This study
FCOV 1-2_1917_3F	ATGGCTTTTTACAAGAGTG	FCOV 1-2_3774_3R	CTATAATCATTGAGCATCGTCTC	1917-3774	ORF1ab (Type I and II)	1857	This study
FCOV 1-2_3497_4F	GAACAGGAAGCTGAACAACC	FCOV 1-2_4451_4R	CAAAAAGCACRGYRTTACCAGG	3497-4451	ORF1ab (Type I and II)	954	This study
FCOV 1-2_4122_5F	CATCCACTTTGTTTCTTAATGC	FCOV 1-2_5370_5R	ACACCRTRRACACAARTTTCATC	4122-5370	ORF1ab (Type I and II)	1248	This study
FCOV 1-2_5127_6F	TCACKCGTAAAACTGCYGGATTTG	FCOV 1-2_6326_6R	AACCACAGRTTRAGGTACTG	5127-6326	ORF1ab (Type I and II)	1199	This study
FCOV 1-2_5896_7F	CTTAGAAAATCTAAGATGCCT	FCOV 1-2_7235_7R	GCACTTACCRATRTCCATAGC	5896-7235	ORF1ab (Type I and II)	1339	This study
FCOV 1-2_6955_8F	CACAAYTAATYGGTAGGCCT	FCOV 1-2_8181_8R	CCAGTTGTCGCCACCGAAGC	6955-8181	ORF1ab (Type I and II)	1226	This study
FCOV 1-2_7626_9F	GTTATTAAGAAYGGYGTTGTTC	FCOV 1-2_8771_9R	TTAGCATATGACTTAATTCTATC	7626-8771	ORF1ab (Type I and II)	1145	This study
FCOV 1-2_8473_10F	CACATTCTTTATGATTGTTTATGC	FCOV 1-2_9841_10R	CACTCTGAAGATTAACACCATAC	8473-9841	ORF1ab (Type I and II)	1368	This study
FCOV 1-2_9632_11F	GAATCTTACAATGCATGGGCC	FCOV 1-2_10942_11R	TCAATRAGATCACTAAGRTCA	9632-10942	ORF1ab (Type I and II)	1310	This study
FCOV 1-2_10560_12F	GGGTTAAYAGRTTTACATGCATG	FCOV 1-2_12052_12R	GCCATACCATTACCGGCACCA	10560-12052	ORF1ab (Type I and II)	1492	This study
FCOV 1-2_11885_13F	ACTCTTAGACGTGGHGCHGTTCT	FCOV 1-2_13502_13R	GTAACACCAGTACTCAAAGCTG	11885-13502	ORF1ab (Type I and II)	1617	This study
FCOV 1-2_13192_14F	CCTAAYTGYTCTGATTGTACA	FCOV 1-2_1698_14R	TCTGGTTCTACCCAACACTT	13192-1698	ORF1ab (Type I and II)	1506	This study
FCOV 1-2_14339_15F	GTACAACTAGTGGTGATGGTAC	FCOV 1-2_16441_15R	GAGACTGTYTTAACAATYTCAGC	14339-16441	ORF1ab (Type I and II)	2102	This study
FCOV 1-2_16100_16F	GGATAATACCYCAAAGAATCAG	FCOV 1-2_18112_16R	GCACCACCATTACAACCTTC	16100-18112	ORF1ab (Type I and II)	2012	This study
FCOV 1-2_17806_17F	AAGATCAATAAAGCTGGTCGCA	FCOV1_19912_17R	CCATTAATRTATGTAAAGAAGCC	17806-19912	ORF1ab, S (Type I)	2106	This study
		FCOV2_19700_17R	AGTACTACCAGGAGCAACAC	17806-19700	ORF1ab, S (Type II)	1946	This study
FCOV1_19664_18F	TGCGTGTTYTGCAYTTRGGA	FCOV1_21610_18R	TCAAATGTRAARTRHAACTG	17806-21610	S (Type I)	2004	This study
FCOV1_21349_19F	TGGTTTYTGYTATCAGATAA	FCOV1_23353_19R	CGTGCACCWAGGTTAAGGG	17806-23353	S (Type I)	2179	This study
FCOV1_23091_20F	GCTATTTGTAATACTGGTGAAAT	FCOV1_25270_20R	CAGCCTCYTCAAAAGTATCACCAA	17806-25270	S, ORF3abc (Type I)	1488	This study
FCOV1_24476_21F	CGTATATGDTRACWCCTCGT	FCOV1_25964_21R	GAACTTCTCAYRAACGGTGCAG	17806-25964	ORF3abc, E (Type I)	1894	This study
FCOV2_19375_18F	GTGTCYAAAGTTGTGGATG	FCOV2_20755_18R	TGCACATGAAATAATAATGG	17806-20755	S (Type II)	1380	This study

FCOV2_20735_19F	CTGGTAATGCAMGKGGTAAACC	FCOV2_22434_19R	ATGARAAAGGACAAGTACCAG	17806-22434	S (Type II)	1699	This study
FCOV2_21854_20F	TGGTTACAATTTCTTTAGCA	FCOV2_23670_20R	CCATTRTAATATTGWGCACA	17806-23670	S, ORF3abc (Type II)	1816	This study
FCOV2_23496_21F	GGTGGTTCTTGGYTAGGAGGT	FCOV2_25203_21R	AGTTACAGCAAAGTATGCACG	17806-25203	S, ORF3abc (Type II)	1707	This study
FCOV2_25034_22F	CCAATTGAAAAAGTGCATGTCCAC	FCOV 1-2_26694_22R	ACAGACCAGCTGAAGTTCCAG	25034-26694	E, M, N (Type I and II)	1660	This study
FCOV 1-2_26561_23F	GCGTTTATGGWGAACGCTA	FCOV 1-2_28223_23R	GAGCGTGACTTTCACTTGATC	26561-28223	M, N,7a (Type I and II)	1662	This study
FCOV 1-2_28061_24F	GTGACAAMTTTCTATGGTGC	FCOV 1-2_29618_24R	ACAATCACTAGATCCAGACG	28061-29618	7ab (Type I and II)	1557	This study

Supplement Table 2. Statistical analysis of the association of FCoV positive rate and demographics and seasonal pattern

Variables	FCoV positive number		
	Number of positive samples	Number of negative samples	*p- value*
**Age**			
Young cats (<6 months)	19	27	
Older cats (> 6 months)	22	92	0.001*
**Sex**			
Male	32	91	0.43
Female	19	70	
**Season**			
Summer (Mar-May)	14	72	
Rainy (Jun-Oct)	29	123	0.124
Winter (Nov- Feb)	18	43	

*Statistical significance, *p-value* <0.01

Supplement Table 3. Genotype identification of positive Thai-FCoVs (n=61) in this study

No	Sample ID	Source	Age	Breed	Sex	Collection Date	Location	Genotype identification of FCoV	
								FCoV-I	FCoV-II
1	CU26329	RS	4 M	N/A	F	21-Jan	Bangkok	+	-
2	CUFIP453	Abdominal Fluid	N/A	N/A	N/A	21-Feb	Bangkok	+	-
3	CUFIP486	Abdominal Fluid	N/A	N/A	F	21-Feb	Bangkok	+	-
4	CUFIP601	Abdominal Fluid	N/A	N/A	N/A	21-Feb	Bangkok	+	-
5	CUFIP606	Abdominal Fluid	N/A	N/A	N/A	21-Feb	Bangkok	+	-
6	CU26828	RS	N/A	N/A	F	21-Mar	Nonthaburi	+	-
7	CU26838	RS	N/A	N/A	F	21-Mar	Nonthaburi	+	-
8	CU26878	RS	7 Y	N/A	M	21-Mar	Bangkok	+	-
9	CU26880	RS	3 Y	N/A	F	21-Mar	Bangkok	+	-
10	CU26881	RS	7 M	N/A	F	21-Mar	Bangkok	+	-
11	CU26882	RS	2 M	N/A	M	21-Mar	Bangkok	+	-
12	CU26952	RS	N/A	DSH	M	21-Apr	Nonthaburi	+	-
13	CU26956	RS	N/A	DSH	F	21-Apr	Nonthaburi	+	-
14	CU26960	RS	2 Y	American Short Hair	M	21-Apr	Nonthaburi	+	-
15	CU26968	RS	11 Y	DSH	F	21-Apr	Bangkok	+	-
16	CU26980	RS	9 Y	DSH	MN	21-Apr	Bangkok	+	-
17	CU26982	RS	N/A	N/A	M	21-Apr	Bangkok	+	-
18	CU27009	RS	4 M	DSH	M	21-Apr	Nonthaburi	+	-
19	CU27013	RS	1 Y	Persian	M	21-Apr	Nonthaburi	+	-
20	CU27468	RS	4 M	DSH	M	21-Jun	Bangkok	+	-
21	CU27528	RS	3 M	Ragdoll	M	Jul-21	Bangkok	+	-
22	CU27529	RS	3 M	DSH	M	Jul-21	Bangkok	+	-
23	CU27541	RS	7 Y	DSH	M	21-Aug	Samut Prakan	+	-
24	CU27694	RS	2 M	DSH	M	21-Aug	Bangkok	+	-
25	CU27697	RS	2 M	DSH	F	21-Aug	Bangkok	+	-
26	CU27698	RS	1 Y	Scottish fold	M	21-Aug	Nonthaburi	+	-
27	CU27704	RS	2 M	N/A	N/A	21-Aug	Bangkok	+	-
28	CU27706	RS	1 Y	DSH	M	21-Aug	Nonthaburi	+	-
29	CU27716	RS	2 Y	DSH	M	21-Aug	Nonthaburi	+	-
30	CU27717	RS	N/A	DSH	F	21-Aug	Nonthaburi	+	-
31	CU27720	RS	12 Y	DSH	F	21-Aug	Bangkok	+	-
32	CU27724	RS	N/A	DSH	M	21-Aug	Nonthaburi	+	-
33	CU27728	RS	3 M	Mixed	M	21-Aug	Bangkok	+	-
34	CU27732	RS	12 Y	DSH	F	21-Aug	Bangkok	+	-
35	CU27740	RS	6 M	DSH	M	21-Aug	Bangkok	+	-
36	CU27761	RS	N/A	DSH	M	21-Aug	Bangkok	+	-
37	CUFIP2175	Abdominal Fluid	N/A	N/A	N/A	21-Aug	Bangkok	+	-
38	CU27766	RS	2 Y	DSH	F	21-Sep	Bangkok	+	-
39	CU27767	RS	3 Y	Scottish fold	M	21-Sep	Bangkok	+	-
40	CU27769	RS	1 Y	Mixed	F	21-Sep	Bangkok	+	-

41	CU27788	RS	3 Y	Ragdoll	F	21-Sep	Bangkok	+	-
42	CUFIP2729	Abdominal Fluid	8 Y	Mixed	M	21-Oct	Bangkok	+	-
43	CUFIP2753	Abdominal Fluid	N/A	N/A	N/A	21-Oct	Samut Prakan	+	-
44	CUFIP2921	Abdominal Fluid	N/A	N/A	N/A	21-Oct	Bangkok	+	-
45	CUFIP2922	Abdominal Fluid	6 M	N/A	F	21-Oct	Bangkok	+	-
46	CUFIP2960	Abdominal Fluid	5 M	Persian	F	21-Oct	Bangkok	+	-
47	CU27829	RS	5 M	British Short Hair	M	21-Oct	Bangkok	+	-
48	CU27832	RS	2 M	Scottish fold	F	21-Oct	Samut Prakan	+	-
49	CUFIP2980	Abdominal fluid	5 M	N/A	M	21-Nov	Bangkok	+	-
50	CUFIP3011	Abdominal Fluid	8 M	N/A	M	21-Nov	Bangkok	+	-
51	CUFIP3021	Thoracic fluid	N/A	N/A	N/A	21-Nov	Bangkok	+	-
52	CUFIP3031	Abdominal fluid	5 M	N/A	M	21-Nov	Bangkok	+	-
53	CUFIP3161	Abdominal fluid	N/A	N/A	F	21-Nov	Bangkok	+	-
54	CUFIP3175	Abdominal fluid	4 M	N/A	M	21-Nov	Bangkok	+	-
55	CUFIP3206	Abdominal fluid	3 M	N/A	M	21-Nov	Bangkok	+	-
56	CUFIP3280	Abdominal fluid	N/A	N/A	M	21-Nov	Nonthaburi	+	-
57	CUFIP3293	Abdominal fluid	6 M	N/A	F	21-Nov	Bangkok	+	-
58	CU28102	RS	2 Y	DSH	M	21-Nov	Samut Prakan	+	-
59	CU28103	RS	3 Y	DSH	M	21-Nov	Samut Prakan	+	-
60	CUFIP3350	Abdominal Fluid	N/A	DSH	M	21-Dec	Bangkok	+	-
61	CUFIP3418	Abdominal Fluid	4 Y	DSH	F	21-Dec	Bangkok	+	-

Supplement Figure 1. Phylogenetic tree based on ORF1ab gene of Thai-FCoVs and reference alphacoronaviruses and betacoronaviruses

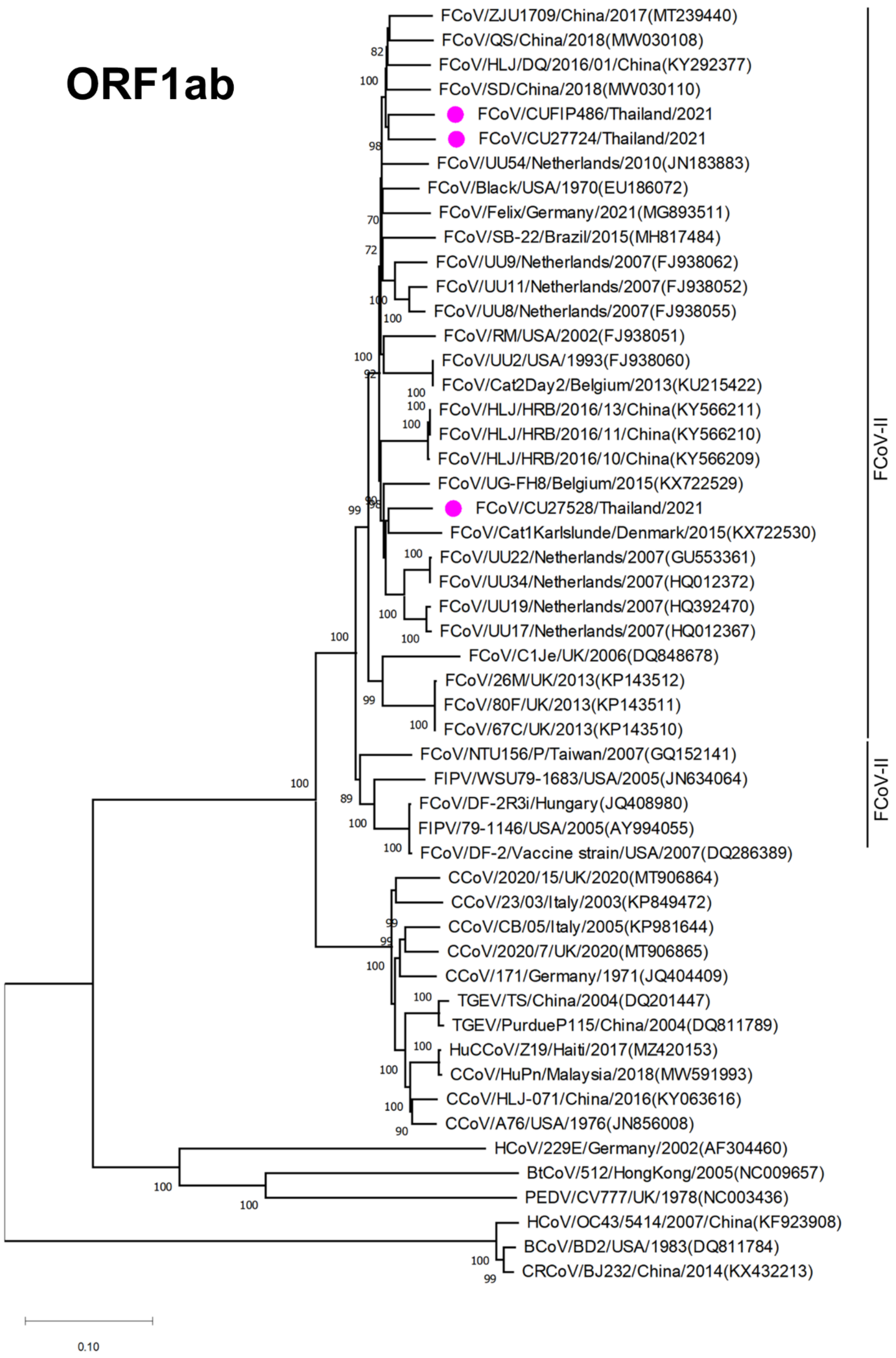

Supplement Figure 2. Phylogenetic tree based on ORF3abc gene of Thai-FCoVs and reference alphacoronaviruses and betacoronaviruses

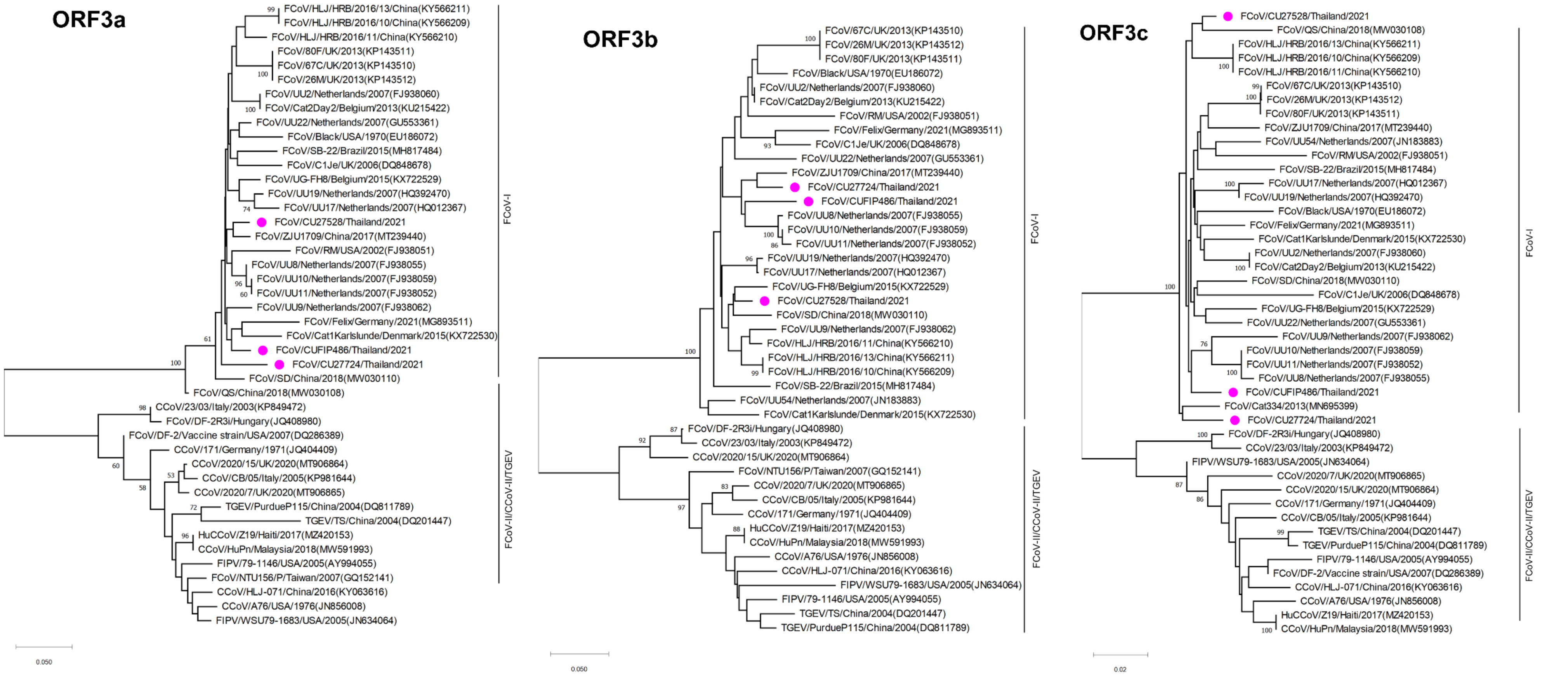

Supplement Figure 3. Phylogenetic tree based on E, M, N gene of Thai-FCoVs and reference alphacoronaviruses and betacoronaviruses

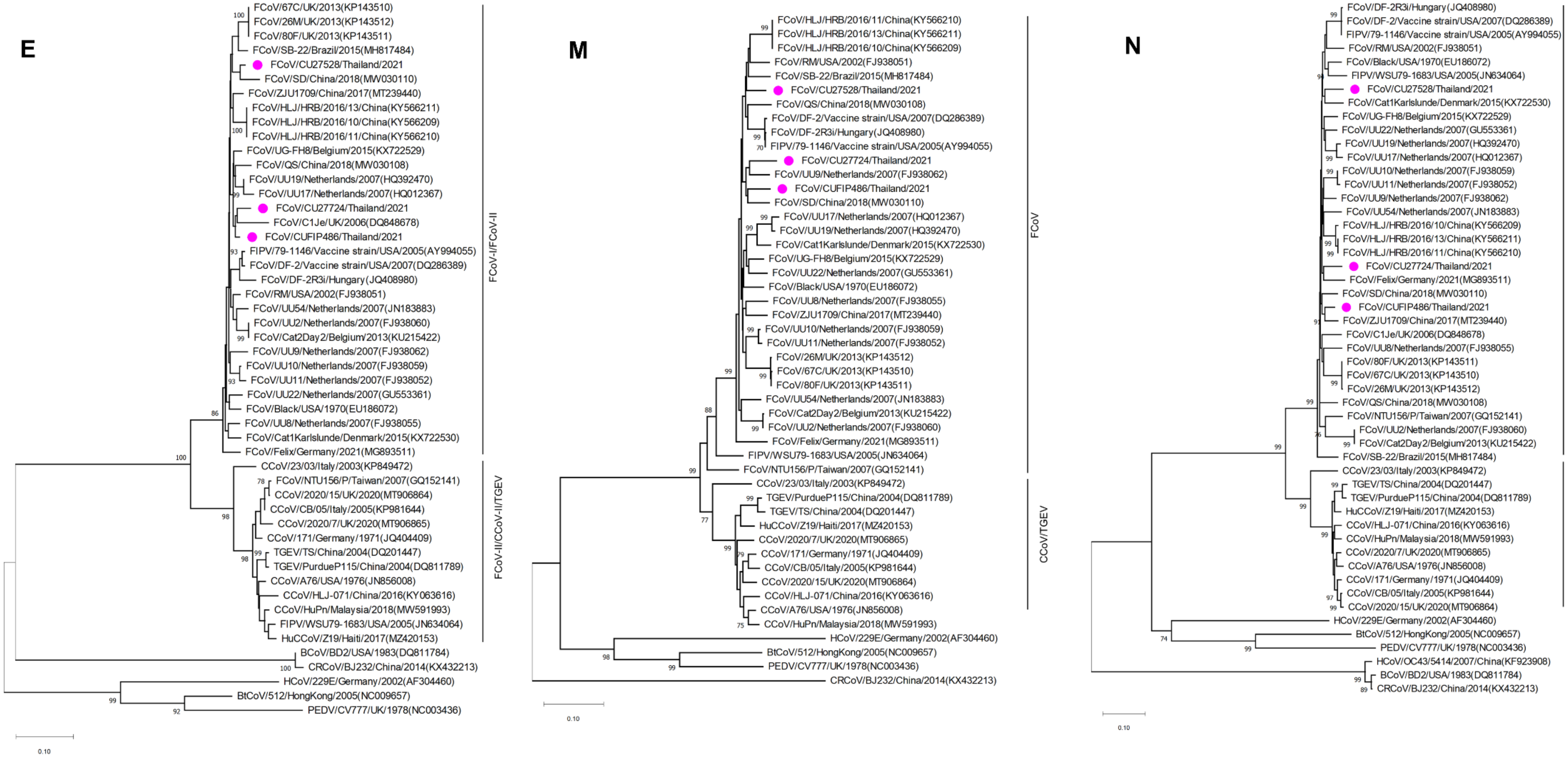

Supplement Figure 4: Phylogenetic tree based on ORF7ab gene of Thai-FCoVs and reference alphacoronaviruses and betacoronavirses

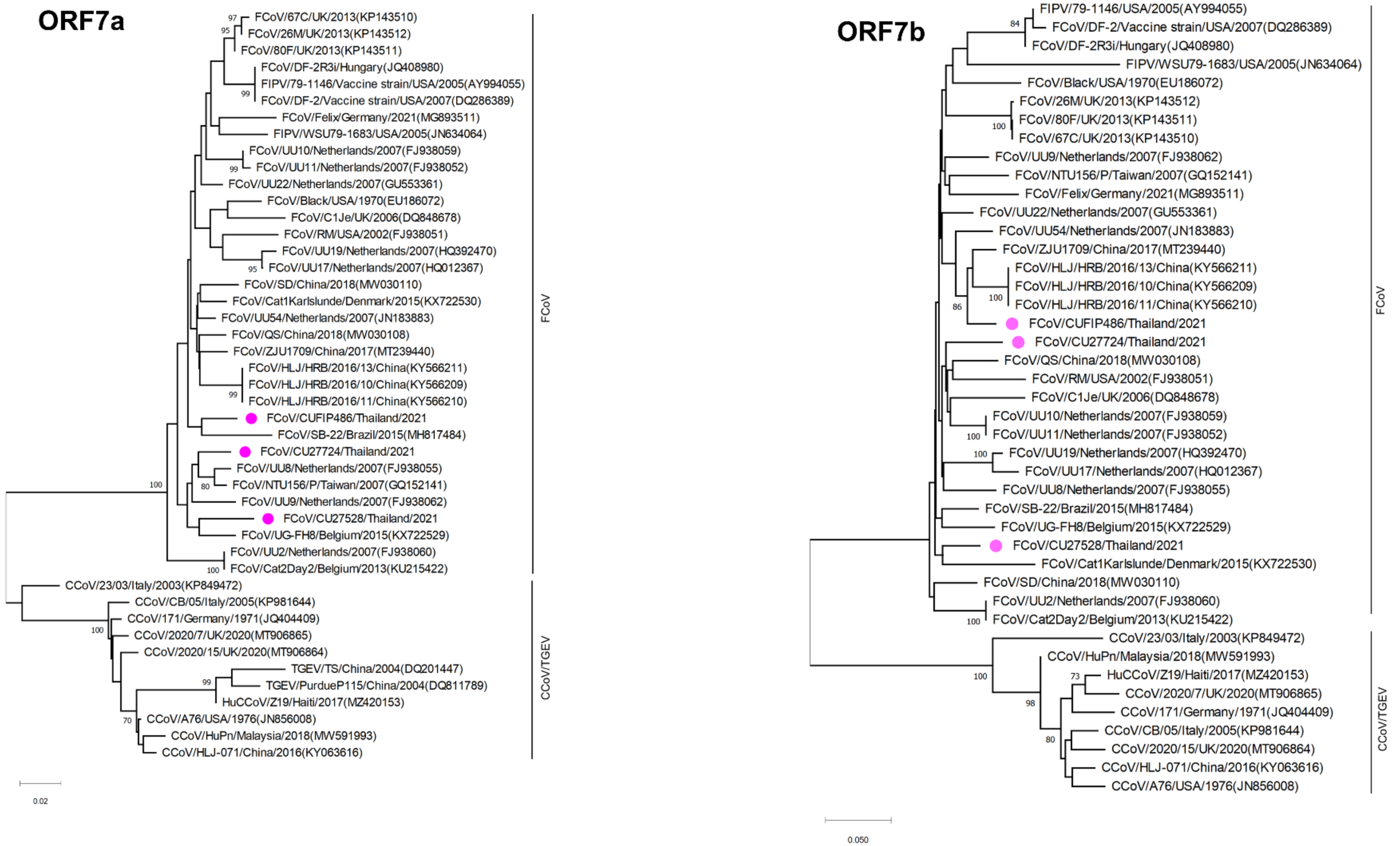

Supplement: Supplementary file 1 [file Data_Sheet_1.pdf]
